# The rise of the longitudinal arch when sitting, standing, and walking: Contributions of the windlass mechanism

**DOI:** 10.1371/journal.pone.0249965

**Published:** 2021-04-08

**Authors:** Freddy Sichting, Florian Ebrecht

**Affiliations:** Department of Human Locomotion, Chemnitz University of Technology, Chemnitz, Germany; Fondazione Santa Lucia Istituto di Ricovero e Cura a Carattere Scientifico, ITALY

## Abstract

The original windlass mechanism describes a one-to-one coupling between metatarsal joint dorsiflexion and medial longitudinal arch rise. The description assumes a sufficiently stiff plantar aponeurosis and absence of foot muscle activity. However, recent research calls for a broader interpretation of the windlass mechanism that accounts for an extensible plantar aponeurosis and active foot muscles. In this study, we investigate the rise of the arch in response to toe dorsiflexion when sitting, standing, and walking to discuss the windlass mechanism’s contributions in static and dynamic load scenarios. 3D motion analysis allowed a kinematic investigation of the rise and drop of the arch relative to the extent of toe dorsiflexion. The results suggest that static windlass effects poorly predict the relationship between arch dynamics and metatarsophalangeal joint motion during dynamic load scenarios, such as walking. We were able to show that toe dorsiflexion resulted in an immediate rise of the longitudinal arch during sitting and standing. In contrast, a decrease in arch height was observed during walking, despite toe dorsiflexion at the beginning of the push-off phase. Further, the longitudinal arch rose almost linearly with toe dorsiflexion in the static loading scenarios, while the dynamic load scenario revealed an exponential rise of the arch. In addition to that, the rate of change in arch height relative to toe motion was significantly lower when sitting and standing compared to walking. Finally, and most surprisingly, arch rise was found to correlate with toe dorsiflexion only in the dynamic loading scenario. These results challenge the traditional perspective of the windlass mechanism as the dominating source of foot rigidity for push-off against the ground during bipedal walking. It seems plausible that other mechanisms besides the windlass act to raise the foot arch.

## Introduction

**Fig 1:**
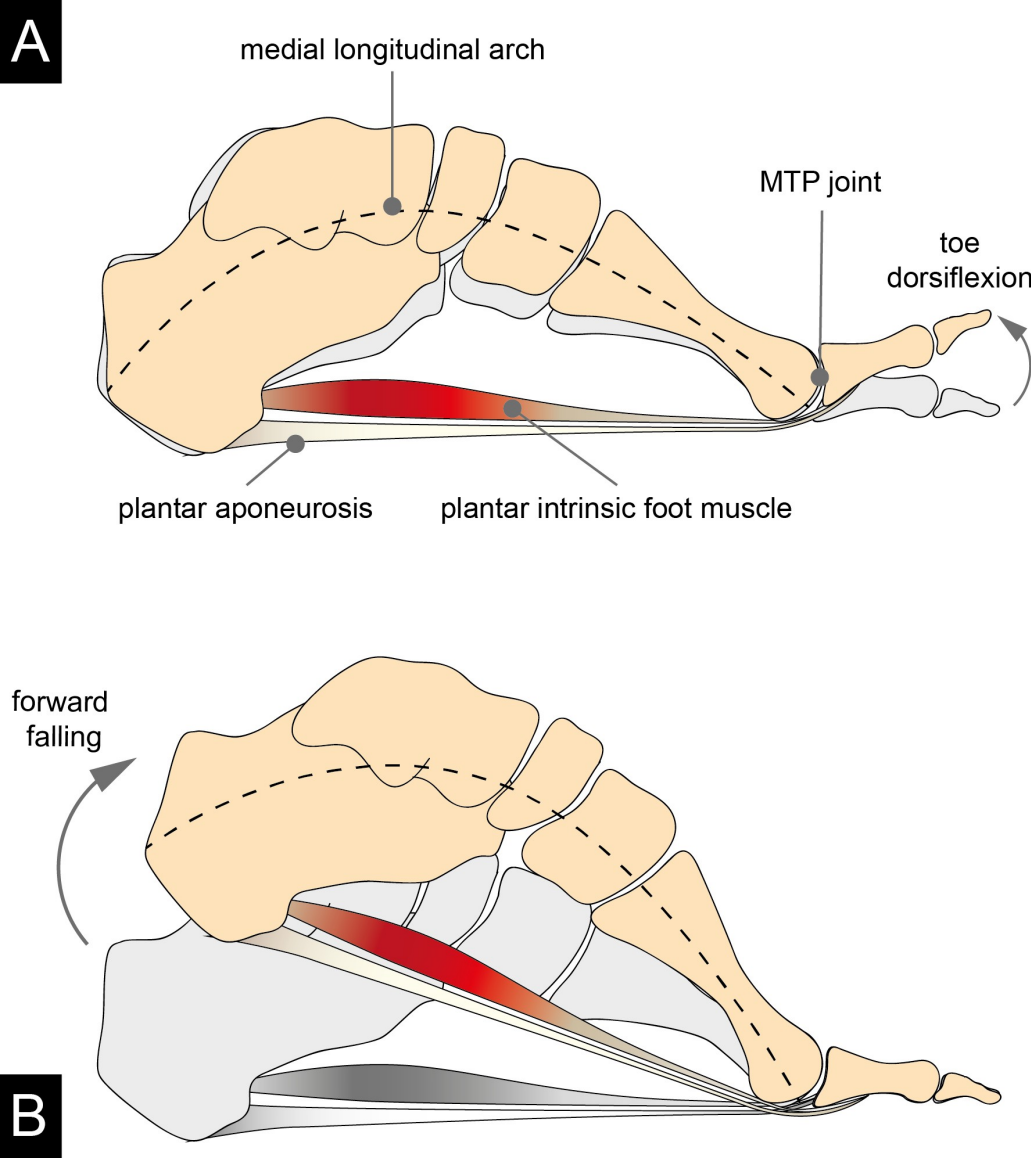
Graphical illustration of the windlass mechanism. (A) depicts the windlass mechanism as a mechanical model that helps explain the foot’s ability to act as an effective and efficient lever. Dorsiflexion of the toes creates tension in the plantar aponeurosis that tends to pull the calcaneus towards the metatarsal heads. This motion creates an upward force in the longitudinal arch. (B) During gait, the windlass mechanism acts at the end of stance when the metatarsal heads and the distal phalanges are the only points of contact with the ground on the trailing leg. Aside from the passive windlass mechanism, however, active plantar intrinsic foot muscles might also be a source of late-stance arch rising.

The human foot has evolved numerous anatomical adaptations in response to the functional demands of bipedal locomotion [1]. These include short toes [2], a prominent longitudinal arch [3], and a well-developed plantar aponeurosis that spans the plantar aspect of the foot from the heel to the toes [4]. Further, the human metatarsal heads are characterized by more dorsally oriented and mediolaterally broader articular surfaces compared to those of our closest relatives, the African apes [5]. During the propulsive phase of walking (from heel lift to toe-off), the dorsally oriented metatarsal heads in the human forefoot are thought to increase the range of dorsiflexion motion at the MTP joints by providing more dorsal articular surface area on which the proximal phalangeal base can slide [6]. From a biomechanical perspective, a wider range of dorsiflexion at the MTP joints helps the foot to act as an effective and efficient lever through the windlass mechanism [7]. Hicks first described the windlass mechanism in 1954 as a one-to-one coupling between metatarsal joint dorsiflexion and medial longitudinal arch rise [7]. According to his description, dorsiflexion of the toes tightens the plantar aponeurosis and, thereby, pulls the calcaneus and metatarsal heads towards each other (Fig 1). Consequently, the longitudinal arch rises. It is thought that the mechanism counters compressive forces from above and stiffens the foot [7].

Since the first description of the windlass, several studies were able to confirm the general mechanism for static load scenarios. In a cadaver study, it was shown that dorsiflexion of the toes results in considerable tension in the plantar aponeurosis that can raise the arch [8]. In an in-vivo experiment, Kappel-Bargas et al. [8] found a highly variable but linear relationship between MTP joint dorsiflexion and arch rise when people were sitting. The linear coupling of MTP joint dorsiflexion and arch deformation was confirmed later using computer simulations [9, 10]. Besides investigations of the direct relationship of MTP joint dorsiflexion and arch rise, the windlass model has also been used to examine the consequences of foot pronation on MTP joint motion. Paton [11] found that greater arch compression during standing (as a representative measure of foot pronation) resulted in decreased toe dorsiflexion. Based on the windlass mechanism, it was argued that the arch’s compression increases the tension in the plantar aponeurosis, which increases the dorsiflexion force under the MTP joint, thereby reducing the ability to dorsiflex the toes passively [11].

While static investigations seem to confirm the original windlass model, dynamic study approaches challenge the one-to-one coupling between arch deformation and MTP joint dorsiflexion. Analyses of the relationship between foot pronation and MTP joint motion during walking [12, 13] are in contrast to Paton’s [11] static findings. Another quasi-dynamic experiment by Welte et al. [14] showed that activation of the windlass seems to increase arch compliance instead of transforming the arch complex into a rigid lever. In a recent follow-up study, Welte et al. [15] provide further evidence that the original windlass mechanism, which assumes an inelastic plantar aponeurosis, seems insufficient to explain arch function during running. Their analysis of arch motion relative to MTP joint dorsiflexion revealed three different windlass mechanism phases, which can be attributed to the extensibility of the plantar aponeurosis. Besides a pure windlass, where MTP joint dorsiflexion is accompanied by a simultaneous arch rise, there is evidence of an inhibited and an enhanced windlass. The terms inhibited and enhanced windlass relate to phases in which the plantar aponeurosis either elongates (inhibited) or shortens (enhanced) during MTP joint dorsiflexion [14]. These findings highlight that the extensibility of the plantar aponeurosis has an important effect on arch dynamics during running. More generally, it indicates that the plantar aponeurosis’s elastic behavior strongly affects the windlass mechanism during dynamic load scenarios. It might partly explain the observed discrepancies between static and dynamic windlass observations.

Another explanation for the mismatch between static and dynamic experiments could be differences in muscle recruitment. Recently published research indicates that the plantar extrinsic and intrinsic foot muscles might also play an essential role in late-stance arch rise and stabilization of the MTP joints [15–17]. Anatomically, the plantar intrinsic foot muscles consist of four layers of muscles which are all located within the foot. In particular, the first two layers, including the abductor hallucis, flexor digitorum brevis, abductor digiti minimi, quadratus plantae and lumbricalis, have muscle configurations that align with the medial and lateral longitudinal arches of the foot [18, 19]. These configurations enable effective flexion of the MTP joints and–like the windlass mechanism–arch rising action by pulling the calcaneus and metatarsal heads towards each other [16]. A recent study by Farris et al. [17] shows that the intrinsic foot muscles respond to passive MTP joint dorsiflexion with bursts of activity, even at relatively low arch compressive loads of 0.5 body weights. Those results let us speculate that foot muscle activity might actively support the windlass mechanism during static double-leg stance. Farris et al. [17] also show that the level of foot muscle activity further increased with increasing arch compressive loads (1.0 and 1.5 body weights). This finding is in line with several previous studies which suggest that the plantar intrinsic foot muscles are substantially active during the propulsive phase when the metatarsal heads and the distal phalanges are the only points of contact with the ground on the trailing leg [20–25]. Likely, the active plantar intrinsic foot muscles balance dorsiflexion moments at the MTP joints [17]. In addition to this stabilizing function, the contracting foot muscles might also be a source of late-stance arch rising. Caravaggi et al. [16] addressed this possibility when investigating the tension produced in the plantar aponeurosis during walking. During the propulsive phase, it was found that the longitudinal arch starts rising quickly despite a declining plantar aponeurosis tension [15]. In the same vein, Fessel et al. [26] found no strict relationship between MTP joint dorsiflexion and plantar aponeurosis tension. Their data show decreasing plantar aponeurosis tension despite an ongoing MTP joint dorsiflexion at the end of stance [26]. Together, these studies indicate that, aside from the passive windlass mechanism, plantar intrinsic foot muscles can actively counteract compressive forces during the propulsive phase.

Considering the potential role of an extensible plantar aponeurosis and active plantar intrinsic foot muscles, we argue that the windlass action during a dynamic load scenario should be different from static load scenarios. Therefore, this study will examine the longitudinal arch’s rise in response to MTP joint dorsiflexion during sitting, standing, and walking. When sitting, the rise of the longitudinal arch in response to MTP joint dorsiflexion should mainly result from the tension produced by the plantar aponeurosis [8–10]. Electromyographic studies indicate that the plantar intrinsic foot muscles of the foot are inactive during sitting [27–29]. MTP joint dorsiflexion should trigger intrinsic foot muscle activity only slightly, if at all [16]. When standing, it remains questionable whether the reported level of intrinsic foot muscle activity [16, 29, 30] can assist the windlass mechanism in raising the arch. In contrast, when walking, the relationship between MTP joint dorsiflexion and arch rise is expected to be more complex due to the stretching and shortening of the plantar aponeurosis and plantar intrinsic foot muscles activity. While previous studies often focused on the windlass mechanism either during static or dynamic tasks, this study provides a test of static and dynamic approaches using an ecological protocol. Considering the potential role of plantar aponeurosis extensibility and plantar intrinsic foot muscles, the direct comparison of the windlass mechanism in static and dynamic load scenarios has two aims: 1. To identify possible differences in the windlass mechanism between static and dynamic tasks. 2. To determine the extent to which a rise of the arch depends on MTP joint motion. According to the original windlass model proposed by Hicks (1954) [6], a higher degree of toe dorsiflexion should result in a more pronounced arch rise.

## Methods

### Participants

The study involved human participants and was approved by the ethics committee at the Chemnitz University of Technology (approval number: V-287-17-FE-Füße-02072018). Fifteen males and ten females (28.2 ± 9.0 years, 71.5 ± 12.7 kg, and 175.8 ± 10.0 cm) gave written consent and participated in this study. All participants were healthy and had no current injuries or conditions that would cause gait abnormalities.

### Experimental treatment

**Fig 2:**
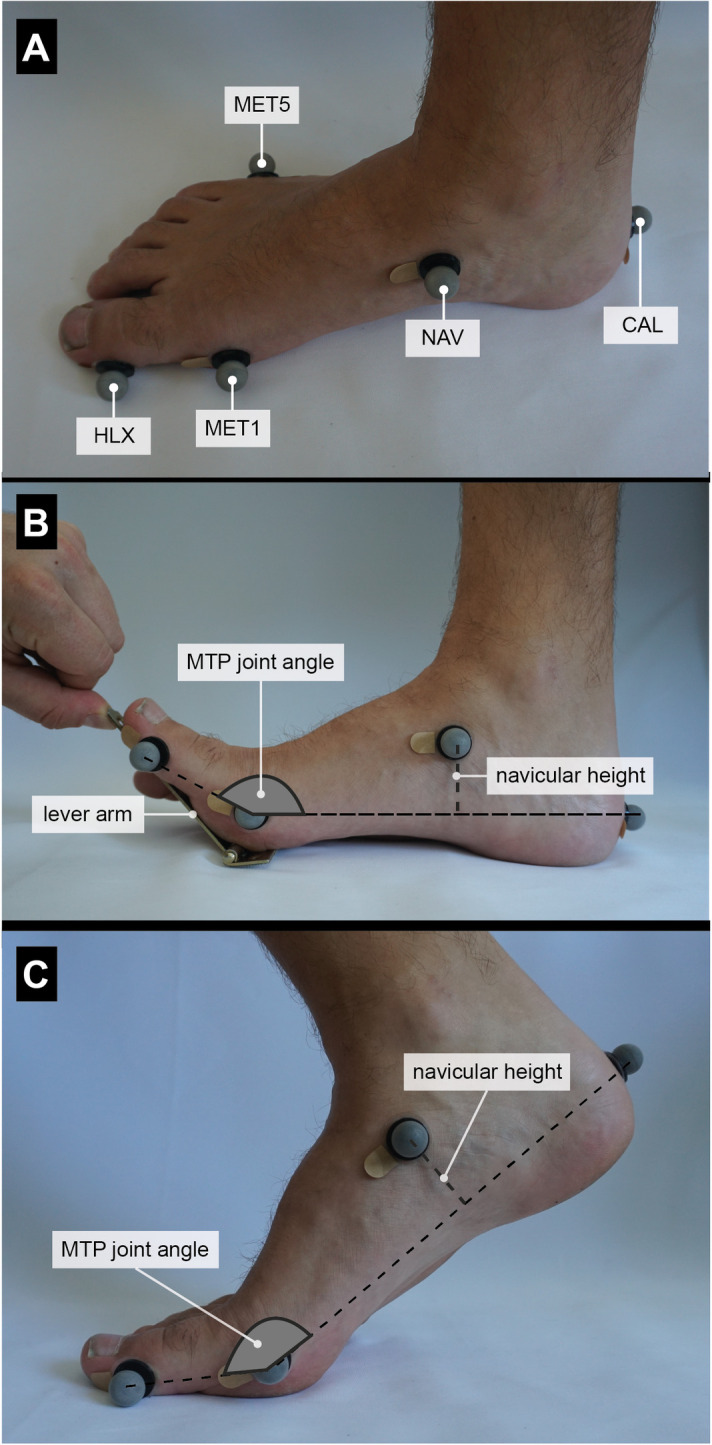
Experimental setup and data analysis. (A) Reflective surface markers were attached at five anatomical landmarks of the right Foot: CAL = posterior calcaneus, MET1 = first metatarsal head, MET5 = fifth metatarsal head, NAV = talo-navicular tuberosity, HLX = medial hallux. (B) A self-built lever arm was used to dorsiflex the first toe. The MTP joint of the toe had to align with the rotational axis of the lever arm. The MTP joint angle was defined by two vectors, which were spanned by the markers CAL, MET1, and HLX. The navicular height was defined as the distance of the NAV marker from the x-y plane (defined by CAL, MET1, and MET5 markers). (C) During walking, change in MTP joint angle and navicular height were recorded between heel-rise and toe-off when the metatarsal heads and the distal phalanges were the only points of contact with the ground on the trailing leg.

We tested the windlass effect in three conditions: sitting, standing, and walking. During sitting, participants were seated with their hip, knee, and ankle each bent to approximately 90°, respectively. During standing, participants were asked to stand in an upright position and distribute their body weight equally on both feet. The center of pressure (COP) was controlled in sitting and standing using a pressure plate (emed® n50, novel gmbh, Munich, Germany) to ensure comparable tension of the plantar aponeurosis among participants [31]. The COP had to be centered under the heel while sitting and in the middle of the foot while standing. Visual feedback was provided to the participants to control the location of the COP relative to the foot. A self-built lever arm was used to dorsiflex the first toe (Fig 2B). The MTP joint of the toe had to align with the rotational axis of the lever arm. The toe was dorsiflexed as far as possible by one examiner pushing on the lever arm. Five consecutive trials were recorded for further data analysis.

Besides sitting and standing, participants had to walk barefoot on a split belt treadmill with force plates under each belt (M-Gait, Motek, Motekforce Link, Amsterdam, Netherlands). Force plates were used to measure the ground reaction forces (GRF) for each step. Participants walked in each condition for a few minutes until they felt comfortable. Walking speed was proportional to leg length as determined by a convention known as a Froude number that follows the principle of dynamic similarity [32]. A Froude number of 0.25 (1.50 ± 0.05 m/s) was chosen for each participant, because it is a comfortable, moderate walking pace. Leg length was measured as the distance from subject’s greater trochanter to the ground (0.92 ± 0.06 m). Kinetic and kinematic data were collected simultaneously across a 20 seconds data collection period. Ten steps were exported for further analysis.

### Acquisition of kinematic and kinetic data

Motion data were captured at 250 Hz using a 10-camera motion analysis system (Vicon Motion Systems Ltd., Oxford, United Kingdom). GRF data were captured synchronously at 1000 Hz. To quantify three-dimensional motions of the foot, five retro-reflective markers (14.0 mm diameter) were placed on the posterior aspect of the calcaneus (CAL), first metatarsal head (MET1), fifth metatarsal head (MET5), tuberosity of the navicular bone (NAV), and lateral side of the Hallux (HLX), as defined elsewhere [33] (Fig 2A).

### Data analysis

Data processing was performed using Vicon Nexus 2.8.1 (Vicon Motion Systems Ltd, UK) and R Studio (R Foundation for Statistical Computing, Vienna, Austria). A recursive fourth-order Butterworth low-pass filter (sitting and standing: 6 Hz cutoff frequency; walking: 10 Hz cutoff frequency) was used to process both kinematic and GRF data. For walking, stance phase (from heel contact to toe-off) was calculated with a 30 N vertical ground reaction force threshold. All kinematic and kinetic data were synchronized and time normalized to stance phase duration for plotting and visual inspection.

#### Joint kinematics

The MTP joint angle was defined through two vectors, which were spanned by the markers CAL, MET1, and HLX. CAL, MET1, and MET5 markers served to define the x-y plane of the foot to calculate navicular height (Fig 2B). The navicular height was then defined as the distance of the NAV marker from the x-y plane [33]. During sitting and standing, the change in MTP joint angle and navicular height were recorded as the toes were dorsiflexed by the examiner pushing on the lever arm. During walking, change in MTP joint angle and navicular height were recorded between heel-rise and toe-off (Fig 2C).

### Statistical analysis

Means and standard deviations (mean ± SDs) were calculated, and a Shapiro-Wilk test of normality was performed for all variables. Further statistical analysis of kinematic measurements between sitting, standing, and walking was performed using a repeated measures ANOVA for normally distributed outcome parameters. When a significant main effect between the conditions was observed, a Bonferroni-adjusted post-hoc analysis was performed. In case of violations of sphericity, the Greenhouse-Geisser adjustment was used. The significance was set at alpha = 0.05 for all tests. Further, a linear regression was used to analyze the correlation between toe dorsiflexion and change in navicular height. All statistical analyses were performed using IBM SPSS Statistics, version 26 (IBM Corporation©, Armonk, New York, USA).

## Results

**Fig 3:**
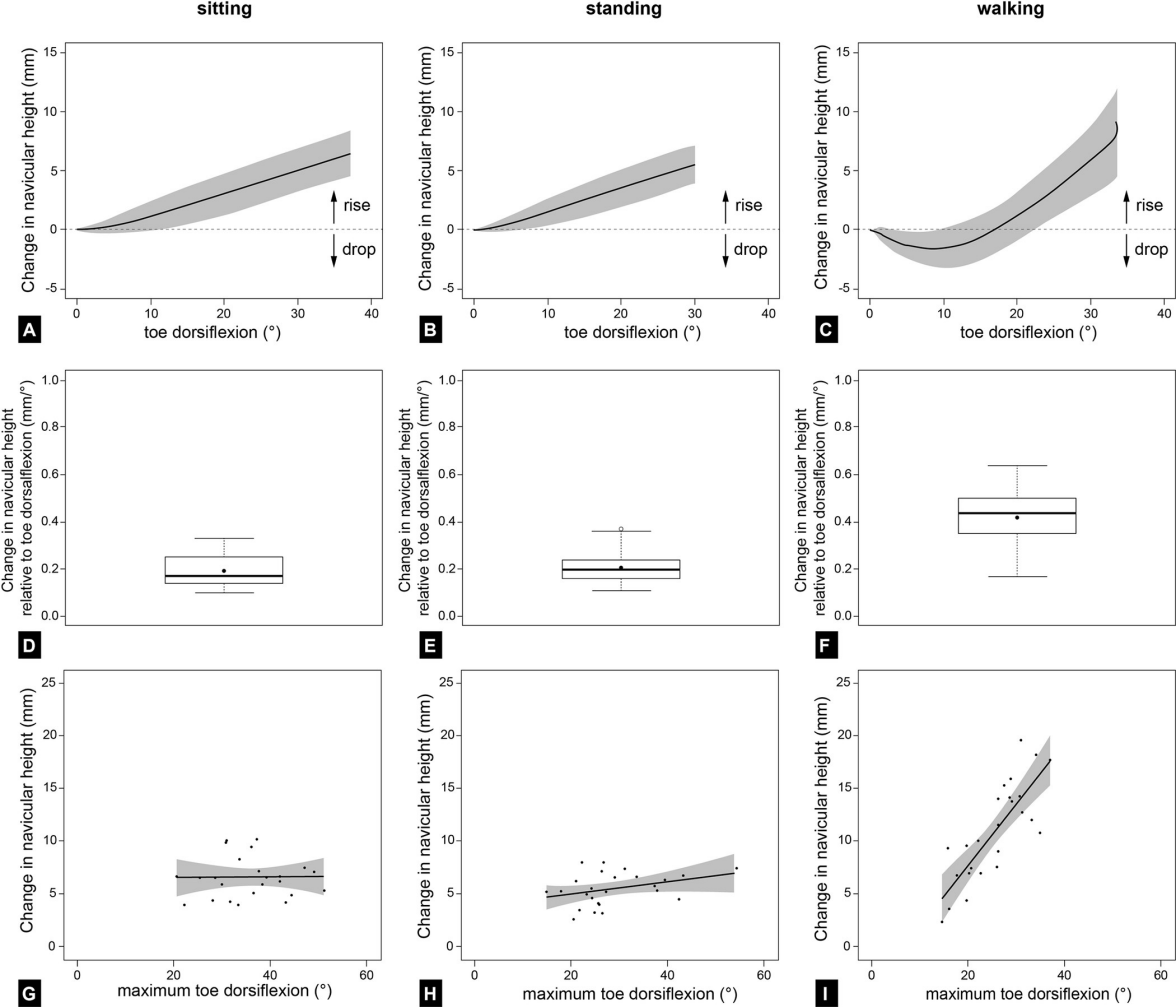
Graphical summary of the results. The results suggest that static windlass effects (sitting and standing) poorly predict the relationship between arch dynamics and metatarsophalangeal joint motion during dynamic load scenarios (walking). (A-C) show the mean changes in navicular height (mm) in relation to toe dorsiflexion (°). (D-F) display the changes in navicular height relative to the toe dorsiflexion (mm/°). Black dots represent the mean value. (G-I) show the linear regression between changes in navicular height (mm) and maximum toe dorsiflexion (°). Each circle represents one participant. The regression line is colored in black. Grey shaded area represents the corresponding confidence interval.

Changes in longitudinal arch motion relative to toe motion were compared by evaluating the rise and drop of the navicular bone. Fig 3A–3I provide an overview of the comparisons between sitting, standing, and walking. Supporting information contains all data underlying the presented findings (S1 and S2 Tables). It can be seen from the data in Fig 3A–3C that dorsiflexion of the toes led to an increase in navicular height (rise). While sitting and standing showed a constant rise of the longitudinal arch with increasing toe angles, the arch did not appear to rise before a toe dorsiflexion angle of 8.51 ± 3.46° during walking. We observed a decrease in navicular height (drop) of 1.55 ± 1.57 mm, followed by a rise of 11.04 ± 4.63 mm. The rise in navicular height during walking was significantly greater compared to sitting and standing (sitting: 6.61 ± 1.92 mm; standing: 5.57 ± 1.55 mm) (p < 0.001, repeated measures ANOVA with Greenhouse-Geisser correction). Interestingly, the Bonferroni-adjusted post hoc analysis also revealed significant differences between sitting and standing (p = 0.002). The total amount of navicular rise was reached at significantly different toe dorsiflexion angles during sitting, standing, and walking (p < 0.001, repeated measures ANOVA). The Bonferroni-adjusted post-hoc analysis revealed significant differences between sitting and standing (37.21 ± 8.03° vs. 29.14 ± 8.97°, p < 0.001), as well as between standing and walking (29.14 ± 8.97° vs. 33.76 ± 5.65°, p = 0.035).

To overcome the differences in total toe dorsiflexion angles and their impact on navicular heights, we further calculated the rate of change in navicular height per degree of toe dorsiflexion (Fig 3D–3F). The comparison between sitting (0.19 ± 0.07 mm/°), standing (0.21 ± 0.07 mm/°), and walking (0.42 ± 0.12 mm/°) showed significant differences (p < 0.001, repeated measures ANOVA with Greenhouse-Geisser correction). The Bonferroni-adjusted post-hoc analyses revealed significant differences between sitting and walking, as well as between standing and walking (p < 0.001 for both comparisons). No statistically significant difference was found between sitting and standing (p = 0.229). To provide more details about arch motion relative to toe rotation, we analyzed the rate of change in navicular height per degree of toe dorsiflexion between 10 to 20° and 20 to 30° toe dorsiflexion angle. The exponential increase in navicular height during walking (10–20°: 0.27 mm/°, 20–30°: 0.50 mm/°) was particularly interesting. Contrary to this, we observed a linear increase in navicular height relative to toe motion during sitting and standing. Notably, the rates of change in navicular height were similar between sitting (10–20°: 0.20 mm/°, 20–30°: 0.20 mm/°) and standing (10–20°: 0.21 mm/°, 20–30°: 0.19 mm/°).

The results of the correlation analysis between toe dorsiflexion and change in navicular height are shown in Fig 3G–3I. What stands out in the figures is the low correlation between the degree of toe dorsiflexion and the resulting change in navicular height during sitting and standing, which was confirmed by our regression analyses (sitting: R < 0.01, F(1,23) = 0.003, p = 0.954), and standing: R = 0.35, F(1,23) = 3.054, p = 0.094). In contrast, we found a significant correlation for walking (R = 0.81, F(1,23) = 43.611, p < 0.001).

## Discussion

The present study was designed to compare the windlass mechanism during static and dynamic load scenarios. We predicted that the windlass action during a dynamic load scenario would be different from a static load scenario. Therefore, we examined the rise of the longitudinal arch in response to MTP joint dorsiflexion in sitting, standing, and walking. Our results indicate that the windlass effects during static load scenarios differ in several aspects from observations in dynamic load scenarios. First, when sitting and standing, MTP joint dorsiflexion resulted in an immediate rise of the longitudinal arch. In contrast, we observed a decrease in arch height despite MTP joint dorsiflexion at the beginning of the push-off phase during walking. Second, while the longitudinal arch rose almost linear with MTP joint dorsiflexion in the static loading scenarios, the dynamic load scenario revealed an exponential rise of the arch. Third, the rate of change in arch height relative to MTP joint motion was significantly lower when sitting and standing than walking. Finally, and most surprisingly, arch rise was found to correlate with MTP joint motion only in the dynamic loading scenario. In general, the results suggest that static windlass effects poorly predict the relationship between arch dynamics and MTP joint motion during dynamic load scenarios, such as walking. It seems plausible that other mechanisms besides the pure windlass act to cause arch rise.

In line with previous studies, dorsiflexion of the MTP joints led to a rise of the arch when sitting and standing [34–36]. The result can be explained by the pulling force of the plantar aponeurosis as described by Hicks [7]. Interestingly, we found no significant difference between sitting and standing, albeit previous research indicates slight activation of plantar intrinsic foot muscles during double-leg stance [29, 30]. Further research reported additional muscle activation due to MTP joint dorsiflexion [16]. Presumably, the level of muscle activity was too low to actively support the arch rise. In contrast to the static load scenarios, the MTP joints’ dorsiflexion did not lead to an immediate rise of the arch during walking. On average, we observed a decline in arch height up to 8.51° toe dorsiflexion, followed by a gradual arch rise. This finding is contrary to the general prediction of the pure windlass. A possible explanation for this might be the plantar aponeurosis’s extensibility and the significantly higher arch compressing forces during walking compared to sitting and standing. It is likely that the plantar aponeurosis further elongates during early MTP joint dorsiflexion [15, 26, 37], which inhibits ongoing arch compression instead of raising the arch [14]. On average, the arch does not rise until 78.1 ± 3.3% of the stance phase, which coincides approximately with the moment of peak arch compressive force [38, 39]. A similar finding was reported recently for running [14].

A further difference in the windlass mechanism between static and dynamic load scenarios is the exponential rise of the arch during walking. The energy-saving behavior of the plantar aponeurosis [40] may partly explain this observation. Several studies have shown that the plantar aponeurosis elongates during midstance and thereby stores energy [14, 15, 26, 37, 41–44]. Later in stance, during propulsion, the plantar aponeurosis shortens and releases the stored energy similar to a spring [40, 43, 44]. Welte et al. [15] observed a similar enhanced arch rise relative to MTP joint motion at the end of stance during running. They argue that the shortening plantar aponeurosis and the windlass mechanism may work in tandem to shorten the arch [14]. We agree with this argument. However, the plantar aponeurosis’s spring-like behavior may not fully explain the exponential rise of the arch. Previous studies indicate that the tension of the plantar aponeurosis decreases at the end of the stance phase (approx. 90% to 100%) [15, 42]. More importantly, the plantar aponeurosis’s length drops close to or even below its initial resting length [14, 15, 26]. Although our current setup cannot measure the remaining strain energy within the plantar aponeurosis, our data let us question whether the elastic energy alone can explain the arch’s exponential rise, especially during late propulsion. Besides the elastic capacities of the plantar aponeurosis, the results could also be related to additional muscle activity. Data from several studies suggest that the plantar intrinsic foot muscles are active during the propulsive phase [21–25]. In a recent study, Farris et al. [17] found that the plantar flexors and plantar intrinsic foot muscles are important sources of tension in the plantar aspect of the foot during push-off and, therefore, could contribute to late-stance arch rising.

The correlation analysis between maximal MTP joint dorsiflexion and corresponding arch rise among all participants in the current study revealed another surprising contrast between the dynamic and static windlass tests. While we found a strong correlation between the amount of MTP joint rotation and the resulting arch rise, no correlations were found in the static load scenarios. Thus, subjects with large MTP joint motions did not show a corresponding large arch rise when sitting and standing. These missing correlations in the static load scenarios seem to reflect a similar observation by Kappel-Bargas et al., who found that subjects varied in the timing of when the windlass mechanism began relative to passive MTP joint rotation [35]. There may be a considerable variation of the pulling force of the plantar aponeurosis in response to toe dorsiflexion. From a clinical perspective, the variation may explain the low sensitivity of the windlass test to diagnose plantar fasciitis. Garceau et al. [44] reported a false negative rate of up to 86.4% in static load scenarios. While we could not measure the pulling force of the plantar aponeurosis directly with our approach, it warrants further study to explain the variability among participants in the static windlass test. Additional testing may consider individual characteristics of the foot, such as foot bone structure, foot dimensions, resting length and mechanical properties of the plantar aponeurosis (and other deep plantar ligaments), passive stiffness of plantar muscles, and joint range of motion. Further, it seems possible that the evoked intrinsic muscle activity due to MTP joint dorsiflexion [16] was powerful enough to support the arch rise in some participants.

It is unfortunate that we were not able to include electromyographic measurements to monitor planar flexor and intrinsic foot muscle activity. A comparison of the level of foot muscle activity evoked by static and dynamic MTP joint dorsiflexion would be of great interest. Another uncontrolled factor in this study is the possibility that the plantar aponeuroses varied among subjects with regard to mechanical properties and anatomical aspects. The plantar aponeurosis is a broad sheet of highly fibrous tissue in which collagen fibers are regularly oriented to span the entire plantar aspect of the foot from the heel to the toes [19]. The prominent central band attaches proximally to the medial tubercle of the calcaneus and fans out distally to attach to the subcutaneous tissue and joint capsules of the second through to the fifth metatarsophalangeal joints, as well as the plantar bases of the corresponding proximal phalanges [45]. According to comparative findings, it is possible to hypothesize that the plantar aponeurosis’s attachment to the bases of the phalanges is highly variable [4], and thereby has the potential to determine the windlass action in static and dynamic load scenarios. Similarly, the mechanical properties can vary among subjects [46, 47], which could also affect the windlass mechanism. Notwithstanding these uncertainties, this study offers valuable insights into the windlass mechanism in static and dynamic load scenarios, which should provoke further discussion of the windlass mechanism statically and in gait.

In general, it seems that static experiments poorly predict the functional role of the windlass mechanism during bipedal walking. The results of this study support the idea that the nonlinear mechanical behavior of the plantar aponeurosis affects the effectiveness of the windlass mechanism [14]. Further, our findings suggest that active muscular contraction could be an additional source of tension along the plantar aspect of the foot that complements the passive windlass mechanism [16]. Our findings support the need for more investigations of the windlass mechanism in dynamic load scenarios to understand its functional importance. Future studies could aim to further untangle the contributions of the pure windlass mechanism, the extensibility of the plantar aponeurosis (and other deep plantar ligaments), and the action of foot muscles as a source of rigidity for push-off against the ground during bipedal walking. Notably, when Hicks described the windlass mechanism for the first time in detail, he noted that "arch-raising is not necessarily the result of action by arch-raising muscles but is a movement that must inevitably occur in every foot, even if dead or paralytic, every time the toes are extended" [7]. He observed that the plantar aponeurosis tenses with dorsiflexion of the toes, which caused the arch to rise. However, he also emphasized that "this […] must not be taken to imply that muscles never have an action upon the arch" [7]. Most likely, the complex nature of the foot with its passive and active structures evolved to act in concert to prevent the foot from pathologies, such as plantar fasciitis.

## Supporting information

S1 Table Summary of the individual results. The results summarize the measured navicular height and metatarsophalangeal (MTP) joint dorsiflexion for each individual who participated in this study.
(PDF)

S2 Table Detailed analysis of arch motion relative to toe rotation. The results summarize data for each individual who participated in this study.
(PDF)

## References

[1] Wright WG, Ivanenko YP, Gurfinkel VS. Foot anatomy specialization for postural sensation and control. J Neurophysiol 2012;107(5):1513–21. 10.1152/jn.00256.2011 22157121 PMC3311689

[2] Rolian C, Lieberman DE, Hamill J, Scott JW, Werbel W. Walking, running and the evolution of short toes in humans. J Exp Biol 2009;212(Pt 5):713–21. 10.1242/jeb.019885 19218523

[3] Holowka NB, Lieberman DE. Rethinking the evolution of the human foot: insights from experimental research. J Exp Biol 2018;221(Pt 17). 10.1242/jeb.174425 30190415

[4] Sichting F, Holowka NB, Ebrecht F, Lieberman DE. Evolutionary anatomy of the plantar aponeurosis in primates, including humans. J Anat 2020. 10.1111/joa.13173 32103502 PMC7309290

[5] Elftman H, Manter J. Chimpanzee and human feet in bipedal walking. Am. J. Phys. Anthropol. 1935;20(1):69–79. 10.1002/ajpa.1330200109

[6] Latimer B, Lovejoy CO. Metatarsophalangeal joints of Australopithecus afarensis. Am. J. Phys. Anthropol. 1990;83(1):13–23. 10.1002/ajpa.1330830103 2221027

[7] Hicks JH. The mechanics of the foot. II. The plantar aponeurosis and the arch. J Anat 1954;88(1):25–30. 13129168 PMC1244640

[8] Carlson RE, Fleming LL, Hutton WC. The biomechanical relationship between the tendoachilles, plantar fascia and metatarsophalangeal joint dorsiflexion angle. Foot Ankle Int 2000;21(1):18–25. 10.1177/107110070002100104 10710257

[9] Cheng H-YK, Lin C-L, Chou S-W, Wang H-W. Nonlinear finite element analysis of the plantar fascia due to the windlass mechanism. Foot Ankle Int 2008;29(8):845–51. 10.3113/FAI.2008.0845 18752786

[10] Cheng H-YK, Lin C-L, Wang H-W, Chou S-W. Finite element analysis of plantar fascia under stretch-the relative contribution of windlass mechanism and Achilles tendon force. J Biomech 2008;41(9):1937–44. 10.1016/j.jbiomech.2008.03.028 18502428

[11] Paton JS. The relationship between navicular drop and first metatarsophalangeal joint motion. J Am Podiatr Med Assoc 2006;96(4):313–7. 10.7547/0960313 16868324

[12] Aquino A, Payne C. Function of the windlass mechanism in excessively pronated feet. J Am Podiatr Med Assoc 2001;91(5):245–50. 10.7547/87507315-91-5-245 11359889

[13] Griffin NL, Miller C, Schmitt D, D’Août K. An investigation of the dynamic relationship between navicular drop and first metatarsophalangeal joint dorsal excursion. J Anat 2013;222(6):598–607. 10.1111/joa.12050 23600634 PMC3666239

[14] Welte L, Kelly LA, Kessler SE, Lieberman DE, D’Andrea SE, Lichtwark GA, et al. The extensibility of the plantar fascia influences the windlass mechanism during human running. Proc Biol Sci 2021;288(1943):20202095. 10.1098/rspb.2020.2095 33468002 PMC7893268

[15] Caravaggi P, Pataky T, Günther M, Savage R, Crompton R. Dynamics of longitudinal arch support in relation to walking speed: contribution of the plantar aponeurosis. J Anat 2010;217(3):254–61. 10.1111/j.1469-7580.2010.01261.x 20646107 PMC2972539

[16] Farris DJ, Birch J, Kelly L. Foot stiffening during the push-off phase of human walking is linked to active muscle contraction, and not the windlass mechanism. J R Soc Interface 2020;17(168):20200208. 10.1098/rsif.2020.0208 32674708 PMC7423437

[17] Farris DJ, Kelly LA, Cresswell AG, Lichtwark GA. The functional importance of human foot muscles for bipedal locomotion. Proc Natl Acad Sci U S A 2019;116(5):1645–50. 10.1073/pnas.1812820116 30655349 PMC6358692

[18] McKeon PO, Hertel J, Bramble D, Davis I. The foot core system: a new paradigm for understanding intrinsic foot muscle function. Br J Sports Med 2015;49(5):290. 10.1136/bjsports-2013-092690 24659509

[19] Gray H. Gray’s anatomy: The anatomical basis of clinical practice. 40th ed. [Edinburgh]: Churchill Livingstone/Elsevier; op. 2008. XXIV, 1551.

[20] Zelik KE, La Scaleia V, Ivanenko YP, Lacquaniti F. Coordination of intrinsic and extrinsic foot muscles during walking. Eur J Appl Physiol 2015;115(4):691–701. 10.1007/s00421-014-3056-x 25420444

[21] Mann R, Inman VT. Phasic activity of intrinsic muscles of the foot. J Bone Joint Surg Am 1964;46:469–81. 14131426

[22] Gray EG, Basmajian JV. Electromyography and cinematography of leg and foot ("normal" and flat) during walking. Anat Rec 1968;161(1):1–15. 10.1002/ar.1091610101 5664082

[23] Kelly LA, Lichtwark G, Cresswell AG. Active regulation of longitudinal arch compression and recoil during walking and running. J R Soc Interface 2015;12(102):20141076. 10.1098/rsif.2014.1076 25551151 PMC4277100

[24] Kelly LA, Lichtwark GA, Farris DJ, Cresswell A. Shoes alter the spring-like function of the human foot during running. J R Soc Interface 2016;13(119). 10.1098/rsif.2016.0174 27307512 PMC4938082

[25] Kelly LA, Farris DJ, Lichtwark GA, Cresswell AG. The Influence of Foot-Strike Technique on the Neuromechanical Function of the Foot. Med Sci Sports Exerc 2018;50(1):98–108. 10.1249/MSS.0000000000001420 28902682

[26] Fessel G, Jacob HAC, Wyss C, Mittlmeier T, Müller-Gerbl M, Büttner A. Changes in length of the plantar aponeurosis during the stance phase of gait—an in vivo dynamic fluoroscopic study. Ann Anat 2014;196(6):471–8. 10.1016/j.aanat.2014.07.003 25113063

[27] Basmajian JV, Bentzon JW. An electromyographic study of certain muscles of the leg and foot in the standing position. Surg Gynecol Obstet 1954;98(6):662–6. 13168829

[28] Basmajian JV, Stecko G. The role of muscles in arch support of the foot: an electromyographic study. J Bone Joint Surg Am 1963;45(6):1184–90. 14077983

[29] Kelly LA, Kuitunen S, Racinais S, Cresswell AG. Recruitment of the plantar intrinsic foot muscles with increasing postural demand. Clin Biomech (Bristol, Avon) 2012;27(1):46–51. 10.1016/j.clinbiomech.2011.07.013 21864955

[30] Kelly LA, Cresswell AG, Racinais S, Whiteley R, Lichtwark G. Intrinsic foot muscles have the capacity to control deformation of the longitudinal arch. J R Soc Interface 2014;11(93):20131188. 10.1098/rsif.2013.1188 24478287 PMC3928948

[31] Hicks JH. The foot as a support. Acta Anat (Basel) 1955;25(1):34–45. 10.1159/000141055 13301184

[32] Alexander RM, Jayes AS. A dynamic similarity hypothesis for the gaits of quadrupedal mammals. Journal of Zoology 1983;201(1):135–52. 10.1111/j.1469-7998.1983.tb04266.x

[33] Eichelberger P, Blasimann A, Lutz N, Krause F, Baur H. A minimal markerset for three-dimensional foot function assessment: measuring navicular drop and drift under dynamic conditions. J Foot Ankle Res 2018;11:15. 10.1186/s13047-018-0257-2 29713385 PMC5907216

[34] Chen D, Li B, Aubeeluck A, Yang Y, Huang Y, Zhou J, et al. Anatomy and biomechanical properties of the plantar aponeurosis: a cadaveric study. PLoS ONE 2014;9(1):e84347. 10.1371/journal.pone.0084347 24392127 PMC3879302

[35] Kappel-Bargas A, Woolf RD, Cornwall MW, McPoil TG. The windlass mechanism during normal walking and passive first metatarsalphalangeal joint extension. Clinical Biomechanics 1998;13(3):190–4. 10.1016/s0268-0033(97)00038-7 11415787

[36] Yawar A, Korpas L, Lugo-Bolanos M, Mandre S, Venkadesan M. Contribution of the transverse arch to foot stiffness in humans. arXiv preprint arXiv:1706.04610 2017.

[37] Gefen A. The in vivo elastic properties of the plantar fascia during the contact phase of walking. Foot Ankle Int 2003;24(3):238–44. 10.1177/107110070302400307 12793487

[38] Kern AM, Papachatzis N, Patterson JM, Bruening DA, Takahashi KZ. Ankle and midtarsal joint quasi-stiffness during walking with added mass. PeerJ 2019;7:e7487. 10.7717/peerj.7487 31579566 PMC6754976

[39] Takahashi KZ, Worster K, Bruening DA. Energy neutral: the human foot and ankle subsections combine to produce near zero net mechanical work during walking. Sci Rep 2017;7(1):15404. 10.1038/s41598-017-15218-7 29133920 PMC5684348

[40] Ker RF, Bennett MB, Bibby SR, Kester RC, Alexander RM. The spring in the arch of the human foot. Nature 1987;325(7000):147–9. 10.1038/325147a0 3808070

[41] Caravaggi P, Pataky T, Goulermas JY, Savage R, Crompton R. A dynamic model of the windlass mechanism of the foot: evidence for early stance phase preloading of the plantar aponeurosis. J Exp Biol 2009;212(Pt 15):2491–9. 10.1242/jeb.025767 19617443

[42] Erdemir A, Hamel AJ, Fauth AR, Piazza SJ, Sharkey NA. Dynamic loading of the plantar aponeurosis in walking. J Bone Joint Surg Am 2004;86(3):546–52. 10.2106/00004623-200403000-00013 14996881

[43] McDonald KA, Stearne SM, Alderson JA, North I, Pires NJ, Rubenson J. The Role of Arch Compression and Metatarsophalangeal Joint Dynamics in Modulating Plantar Fascia Strain in Running. PLoS ONE 2016;11(4):e0152602. 10.1371/journal.pone.0152602 27054319 PMC4824348

[44] Wager JC, Challis JH. Elastic energy within the human plantar aponeurosis contributes to arch shortening during the push-off phase of running. J Biomech 2016;49(5):704–9. 10.1016/j.jbiomech.2016.02.023 26944691

[45] Bojsen-Moller F, Flagstad KE. Plantar aponeurosis and internal architecture of the ball of the foot. J Anat 1976;121(Pt 3):599. 1018010 PMC1231749

[46] Kogler GF, Veer FB, Verhulst SJ, Solomonidis SE, Paul JP. The effect of heel elevation on strain within the plantar aponeurosis: in vitro study. Foot Ankle Int 2001;22(5):433–9. 10.1177/107110070102200513 11428764

[47] Pavan PG, Stecco C, Darwish S, Natali AN, Caro R de. Investigation of the mechanical properties of the plantar aponeurosis. Surg Radiol Anat 2011;33(10):905–11. 10.1007/s00276-011-0873-z 21947015

