# Supplementary material for: The rise of the longitudinal arch when sitting, standing, and walking: Contributions of the windlass mechanism
Source: PLoS One. 2021 Apr 8;16(4):e0249965. doi: 10.1371/journal.pone.0249965 (PMC8031382; doi:10.1371/journal.pone.0249965)
Supplement: S1 Table — The results summarize the measured navicular height and metatarsophalangeal (MTP) joint dorsiflexion for each individual who participated in this study. (PDF) [file pone.0249965.s001.pdf]

	Max. toe dorsiflexion (°)			Absolute change in NAV height (mm)			Toe dorsiflexion (°)			Absolute change in NAV height relative to toe dorsiflexion (mm/°)		
**Participant**				**sitting**	**standing**	**walking**	**sitting**	**standing**	**walking**	**sitting**	**standing**	**walking**
**1**	36.24	24.60	30.09	3.99	3.29	9.45	33.41	24.60	15.86	0.12	0.13	0.60
**2**	39.37	29.65	29.20	9.48	6.64	10.13	36.17	28.80	22.15	0.26	0.23	0.46
**3**	28.44	21.43	31.64	4.40	3.52	13.87	28.02	21.43	29.06	0.16	0.16	0.48
**4**	38.24	22.25	22.69	10.23	8.08	3.63	37.21	21.96	16.06	0.27	0.37	0.23
**5**	53.38	54.10	34.99	5.36	7.50	10.84	51.14	53.61	34.99	0.10	0.14	0.31
**6**	30.96	15.08	36.45	10.06	5.28	15.40	30.86	14.65	27.61	0.33	0.36	0.56
**7**	42.10	42.99	41.48	6.65	6.81	17.80	42.00	42.99	37.18	0.16	0.16	0.48
**8**	48.45	31.45	38.98	7.54	7.51	14.31	47.11	30.85	30.87	0.16	0.24	0.46
**9**	45.94	38.08	39.73	6.21	5.86	2.43	42.05	37.09	14.63	0.15	0.16	0.17
**10**	31.67	25.62	35.70	4.26	4.14	19.69	31.67	25.41	30.92	0.13	0.16	0.64
**11**	43.75	24.99	38.39	4.21	2.67	16.01	43.23	20.27	28.92	0.10	0.13	0.55
**12**	30.24	26.52	28.83	6.56	7.25	9.68	28.57	26.14	19.89	0.23	0.28	0.49
**13**	38.30	39.25	28.47	7.22	6.37	7.62	37.60	39.19	26.01	0.19	0.16	0.29
**14**	33.60	20.79	36.89	8.30	6.34	14.16	33.60	20.79	26.32	0.25	0.30	0.54
**15**	30.76	26.55	35.58	9.95	8.08	14.25	30.76	26.55	28.69	0.32	0.30	0.50
**16**	25.44	28.28	32.54	6.57	5.29	7.51	25.44	26.94	20.62	0.26	0.20	0.36
**17**	38.07	29.51	40.86	5.06	3.26	18.31	36.53	26.07	34.14	0.14	0.13	0.54
**18**	46.15	42.08	40.56	4.94	4.55	12.10	44.40	42.08	33.29	0.11	0.11	0.36
**19**	30.07	23.98	29.84	5.93	5.62	7.00	30.07	23.98	22.70	0.20	0.23	0.31
**20**	49.56	37.53	36.00	7.09	5.37	12.77	49.17	37.53	31.37	0.14	0.14	0.41
**21**	38.30	33.33	37.46	8.13	6.68	9.12	36.59	33.33	26.40	0.22	0.20	0.35
**22**	27.75	25.63	26.47	3.97	4.12	6.82	22.13	25.63	17.76	0.18	0.16	0.38
**23**	22.32	17.65	20.25	6.65	5.36	7.07	20.64	17.65	20.25	0.32	0.30	0.35
**24**	40.42	24.11	37.65	5.90	4.71	11.55	38.42	24.11	26.35	0.15	0.20	0.44
**25**	40.83	22.94	33.36	6.59	5.02	4.51	39.26	22.94	19.79	0.17	0.22	0.23
