# Supplementary material for: The rise of the longitudinal arch when sitting, standing, and walking: Contributions of the windlass mechanism
Source: PLoS One. 2021 Apr 8;16(4):e0249965. doi: 10.1371/journal.pone.0249965 (PMC8031382; doi:10.1371/journal.pone.0249965)
Supplement: S2 Table — The results summarize data for each individual who participated in this study. (PDF) [file pone.0249965.s002.pdf]

	For sitting: NAV height (mm) at toe dorsiflexion of…			For standing: NAV height (mm) at toe dorsiflexion of…			For walking: NAV height (mm) at toe dorsiflexion of…		
**Participant**	**10°**	**20°**	**30°**	**10°**	**20°**	**30°**	**10°**	**20°**	**30°**
**1**	0.26	1.04	2.94	0.82	1.89	NA	-0.20	2.09	8.33
**2**	0.80	2.93	5.91	0.76	3.47	NA	-2.46	2.37	NA
**3**	1.52	3.10	NA	1.77	3.46	NA	0.26	4.90	10.28
**4**	1.81	4.74	7.88	3.22	7.26	NA	0.55	2.29	NA
**5**	0.54	1.90	3.68	1.26	2.70	4.11	0.21	2.87	7.34
**6**	3.18	6.54	9.76	3.24	NA	NA	-3.38	-0.30	5.95
**7**	1.02	2.65	4.47	1.79	3.60	5.07	-0.78	2.60	7.36
**8**	0.82	2.22	4.51	1.82	4.67	7.09	-4.49	-1.30	3.55
**9**	-0.18	1.59	3.11	0.46	2.41	4.44	0.47	-0.74	-0.82
**10**	1.14	2.42	4.15	1.87	3.53	NA	-2.00	3.52	10.47
**11**	0.28	1.02	2.41	-0.09	1.60	NA	-1.08	1.38	6.15
**12**	0.72	3.08	6.34	2.13	5.59	NA	-2.62	1.29	NA
**13**	1.49	3.64	5.64	1.36	3.06	4.90	1.10	4.49	NA
**14**	3.46	5.85	7.75	3.89	6.15	NA	-3.14	-0.91	4.47
**15**	3.37	6.63	9.64	3.87	6.66	NA	-3.30	0.41	6.02
**16**	2.07	4.75	NA	1.08	3.48	NA	-1.40	-0.46	3.56
**17**	0.19	1.48	3.34	0.07	1.12	NA	-3.62	0.54	6.24
**18**	0.31	1.25	2.61	0.62	1.49	2.85	-0.91	1.42	5.36
**19**	2.58	4.38	5.93	2.56	4.64	NA	-1.40	1.32	NA
**20**	0.58	2.04	3.96	0.99	2.27	4.02	-0.53	3.18	7.52
**21**	0.61	2.53	5.64	0.49	2.70	4.90	-1.41	-0.05	3.73
**22**	0.02	1.61	NA	1.56	3.22	NA	-1.83	0.59	NA
**23**	1.46	5.36	NA	1.74	NA	NA	0.93	6.06	NA
**24**	0.50	2.21	4.01	2.09	3.99	NA	-2.43	-0.90	3.91
**25**	0.68	2.53	4.47	1.12	3.87	NA	-1.79	-1.64	0.76
